# HTLV-1 bZIP factor perturbs immune response to the pathogens in vivo by inhibiting IFN-gamma production

**DOI:** 10.1186/1742-4690-8-S1-A102

**Published:** 2011-06-06

**Authors:** Kenji Sugata, Yorifumi Satou, Jun-ichirou Yasunaga, Kisato Nosaka, Masao Matsuoka

**Affiliations:** 1 Laboratory of Virus Control, Institute for Virus Research, Kyoto University, Kyoto, Japan; 2 Department of Hematology, Kumamoto University School of Medicine, Japan

## 

HTLV-1 carriers and the patients with adult T-cell leukemia (ATL) frequently suffer from the opportunistic infections. Although it has been known that HTLV-1 infection evokes cell-mediated immune deficiency, its mechanism remains unknown. HTLV-1 bZIP factor (HBZ) is encoded in the minus strand of HTLV-1, which is constitutively expressed and involved in the proliferation of HTLV-1-infected cells. In this study, we investigated the role of HBZ in immunodeficiency in a mice model. HBZ transgenic (HBZ-Tg) mice were infected with HSV-2 and Listeria monocytogenes and evaluated for cellular immunity to those pathogens. The clearance of both pathogens was impaired in HBZ-Tg mice compared with the non-transgenic littermate. In the both infection, production of IFN-gamma in CD4+ T cells was significantly reduced in HBZ-Tg mice. In addition, ectopic expression of HBZ in primary human CD4+ T cells impaired IFN-gamma production in vitro. As the molecular mechanisms, we found that HBZ suppressed transcription of IFNG promoter. Our results suggest that HBZ plays a crucial role for cellular immunodeficiency in HTLV-1-infected subjects.

